# The death of King Charles XII of Sweden revisited

**DOI:** 10.1093/pnasnexus/pgac234

**Published:** 2022-10-11

**Authors:** Juho-Antti Junno, Markku Niskanen, Heli Maijanen, Jaakko Niinimäki, Alina Junno, Petteri Oura

**Affiliations:** Department of Archaeology, Faculty of Humanities, University of Oulu, FI-90014 Oulu, Finland; Department of Anatomy, Medical Research Center Oulu, Oulu University Hospital and University of Oulu, FI-90014 Oulu, Finland; Department of Archaeology, Faculty of Humanities, University of Oulu, FI-90014 Oulu, Finland; Department of Archaeology, Faculty of Humanities, University of Oulu, FI-90014 Oulu, Finland; Faculty of Medicine, Research Unit of Medical Imaging, Physics and Technology, University of Oulu, FI-90014 Oulu, Finland; Department of Diagnostic Radiology, Oulu University Hospital, Kajaanintie 50, 90220 Oulu, Finland; Department of Archaeology, Faculty of Humanities, University of Oulu, FI-90014 Oulu, Finland; Faculty of Medicine, Research Unit of Medical Imaging, Physics and Technology, University of Oulu, FI-90014 Oulu, Finland; Department of Forensic Medicine, University of Helsinki, Yliopistonkatu 4, 00100 Helsinki, Finland; Forensic Medicine Unit, Finnish Institute for Health and Welfare, FI-00271 Helsinki, Finland

## Abstract

The death of King Charles XII of Sweden has remained as a mystery for more than three centuries. Was he assassinated by his own men or killed by the enemy fire? Charles was killed by a projectile perforating his skull from left to right. In this study, we utilized a Synbone ballistic skull phantom and modern radiological imaging to clarify the factors behind the observed head injuries. We examined whether a musket ball fired from the enemy lines would be the most potential projectile. Our experiments with a leaden 19.5 mm musket ball demonstrated that at velocities of 200 to 250 m/s, it could cause similar type of injuries as observed in the remains of Charles . The radiological imaging supported the theory that the projectile was not a leaden but of some harder metal, as we could detect remnants of lead inside the wound channel unlike in Charles’ case. In addition, our experiments showed that a 19.5mm musket ball  produces max. 17mm hole into a felt material  . The main evidence supporting 19.5 mm projectile size has been a 19-19.5mm bullet hole in a hat that Charles was wearing during his death. Additional experiments with a 25.4 mm steel ball produced approximately 20 mm hole in the felt. As our musket ball experiments also resulted in considerably smaller cranial injuries than those in Charles’ case, we can conclude that the deadly projectile wasn’t leaden and was more than 19.5 mm in diameter, potentially an iron cartouche ball that was shot from the enemy lines.

Significance statement The death of King Charles XII of Sweden has been a topic of great interest and debate for more than 300 years. Clear consensus about the factors such as deadly projectile involved with his death are still lacking although three autopsies and latest research techniques such as DNA-analyses are experimented to enlighten the case.

## Introduction

**Fig. 1.**
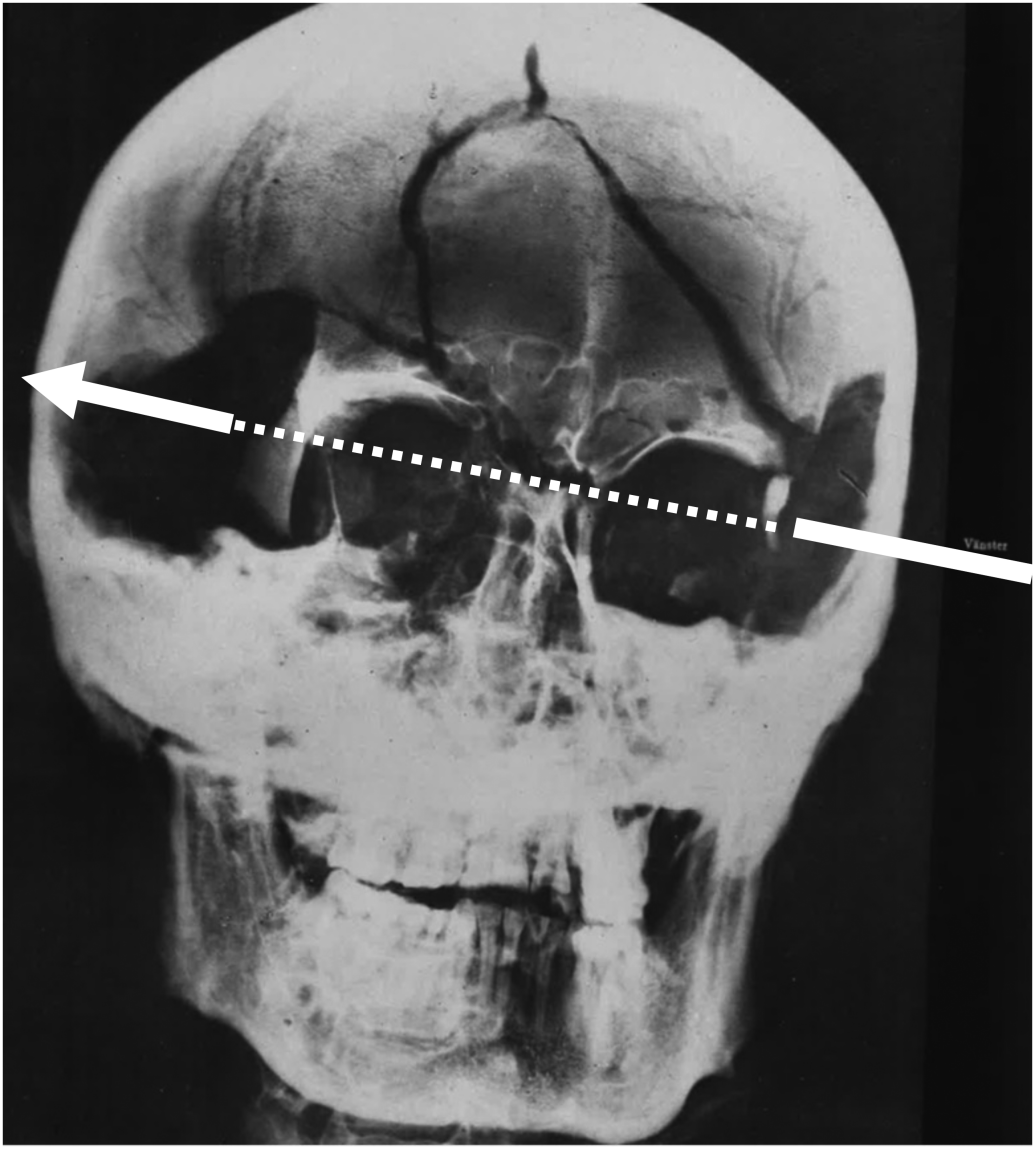
A radiograph from the latest autopsy (1) showing the wound channel and direction of the projectile.

In November 1718, during the siege of Fredriksten, King Charles XII of Sweden was killed by a projectile fully perforating his skull. For 300 years, the exact course of events has remained as a mystery. Several autopsies (in 1746, 1859, and 1917) have concluded that Charles died of a single projectile travelling through his head from left to right (Fig. 1). Undisputed evidence about the projectile is, however, lacking (1).

Although several theories suggest assassination [e.g. (2, 3)], a more natural explanation would be enemy fire (4). Many of these theories and even relatively recent scientific work are flavored with folklore and speculation [e.g. (3)] even though already Ahnlund (5) criticized the use of folklore in this particular case.

The most recent autopsy was performed in 1917 (1). Detailed external and internal examinations revealed major soft tissue and bone damage on the left and right sides of the head, corresponding to entrance and exit sites, respectively. Soft tissue damage on the left side took an oval shape with axes 47 mm and 37 mm. On the right side, soft tissue damage followed the shape of a circular segment, with a basis 30 mm and height 20 mm. The bony defect on the left was relatively circular with diameters 55 to 63 mm, while the defect on the right had an uneven shape and larger size with diagonals 55 to 75 mm.

**Fig. 2.**
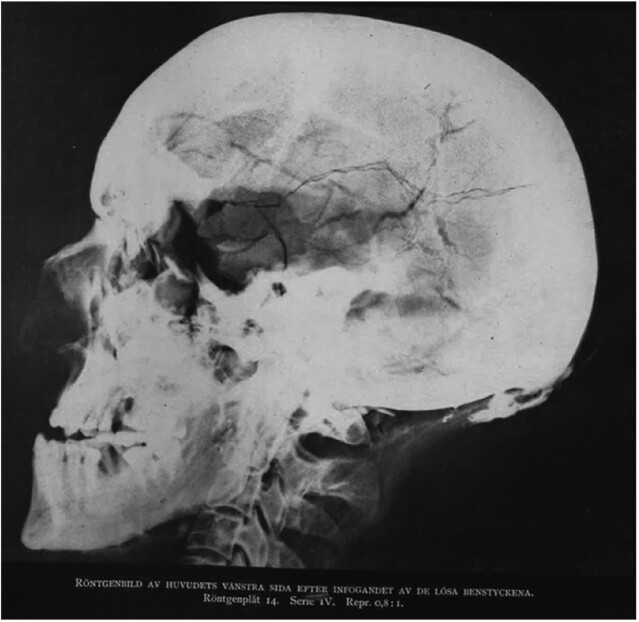
Radiographs from the latest autopsy performed in 1917 (1) show no traces of lead in the wound channel or elsewhere in the cranium.

The 1917 autopsy was supplemented with radiological imaging. As radiographs showed no traces of lead (Fig. 2), it was argued that the projectile was of some harder metal [e.g. (3)]. Iron, for example, was used in cartouche shots of cannons. In addition, a so-called bullet-button hypothesis (6) suggesting that the projectile was originally a button shot from a musket, has gained popularity among scholars [e.g. (7)]. Even DNA-analyses have been performed on the button to enlighten its role as a deadly projectile (8).

Charles died late in the evening while inspecting trench digging near the fortress. There is no detailed information about his exact position; however, the closest distance to enemy lines was estimated to be 175 m [e.g. (9)]. At that time muskets could produce muzzle velocity of more than 400 m/s [e.g. (10, 11)] and from that distance the velocity would have decreased roughly by 200 m/s.

Several studies have aimed to enlighten the details regarding the course of events. Using ballistic calculations, Grenander (4) concluded that Charles was killed by the enemy fire. The experiments of Hultkvist (9) suggested that the projectile had the same diameter as a musket ball and was shot by the Swedish.

The projectile that killed Charles also pierced his felt hat leaving a hole of 19 to 19.5 mm in diameter (1). As entrance and exit tissue defects cannot confirm exact projectile size [e.g. (12)], the hole in the hat has been interpreted as a direct measure of the projectile diameter. The hat has thus been taken as a direct piece of evidence supporting the bullet-button hypothesis as the hole and the famous button are relatively similar in diameter (6).

In this experimental study, we approached the death of King Charles XII of Sweden in a multimodal manner with modern forensic methods to enlighten the course of events. We hypothesized that latest ballistic phantoms aided by modern radiological imaging [e.g. (13)] would provide us with new detailed information about the material, velocity, and size of the projectile.

## Materials and Methods

### Shooting experiment

Altogether 12 shots were fired in this experiment (Table 1). Four included ballistic skull phantoms and eight tested different projectiles and velocities on felt hole measurements. Synbone generic 5-mm-thick 190-mm sphere with artificial skin served as the ballistic skull phantom. The Synbone sphere is widely recognized as a substitute of human head in ballistic and forensic experiments , (14)]. Spheres were filled with 10% gelatin prepared according to Jussila (15). We attached two layers of 4 mm thick industrial felt in front and behind the sphere to replicate the hat of Charles XII.

**Table 1.** Table 1 shows the metric data obtained from each experiment.

Experiment number	velocity (m/s)	Bullet weight (g)	Weight reduction (g)	Entrance wound (mm)	Exit wound (mm)	Hole size (felt)
1 Synbone	193	43,2	0,6	21	25 to 30	12 mm
2 Synbone	194	43,4	0,5	22	25 to 35	14 mm
3 Synbone	265	43,5	0,8	50 to 55	20 (50)	17 mm
4 Synbone	152	43,4	0,4	20	25	11 mm
5 Felt 4 mm	227					16 mm
6 Felt 4 mm	258					15 mm
7 Felt wool	236					13 mm
8 Felt wool	178					15 mm
9 Cannon	380					20 mm
10 Cannon	n/a					21 mm
11 Cannon	n/a					21 mm
12 Cannon	214					20 mm

First eight experiments are with a 19.5-mm leaden musket ball and last four with a 25.4-mm steel ball. First four experiments included Synbone phantom + felt and last eight just felt.

Only one type of projectile was used in skull phantom experiments. A round musket ball (diameter 19.5 mm) was selected based on previous literature and especially the 19 to 19.5 mm hole in the hat of Charles (1, 9). Musket balls were casted from pure lead and their weight ranged from 43.2 to 43.5 g.

Remington SP-10 shotgun with 30” barrel length was used to shoot musket balls. We aimed to test three speed categories according to previous research on potential projectile velocity in impact (4, 9). We were most interested in the speed of approximately 200 m/s as that would be a realistic velocity for a musket ball fired from the fortress of Fredriksten, some 200 m from the death scene. Three different powder loads were selected to achieve musket ball velocities of 150, 200, and over 250 m/s.

**Fig. 3.**
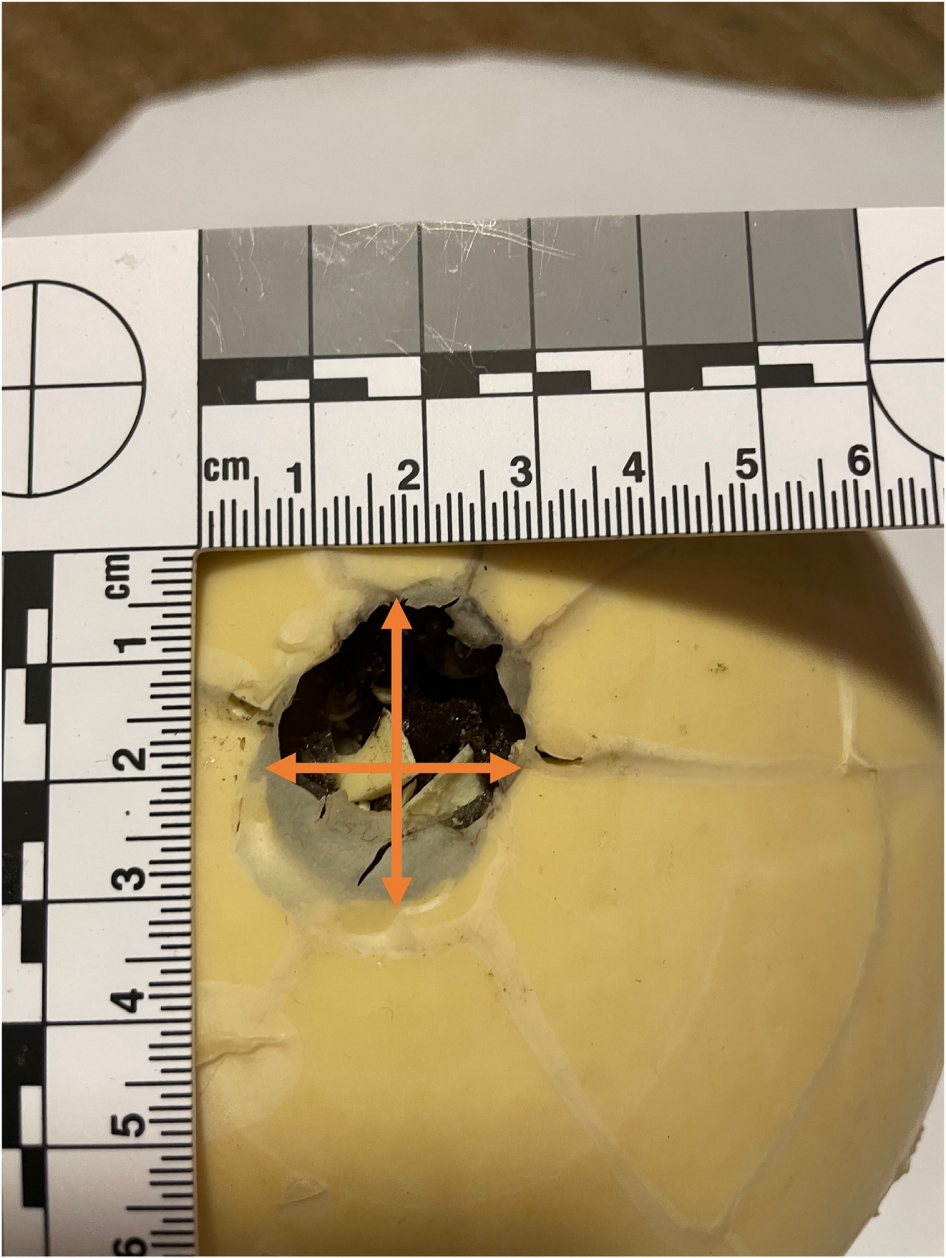
The diameter of the entrance and exit wounds were measured using standard digital calipers. Two perpendicular measurements were taken and their mean was then calculated.

Test firing was performed at an enclosed shooting range following adequate safety measures. Skull phantoms were positioned on a solid, wooden platform. Shots were fired from a distance of 5 m and ball velocity measured with a Caldwell chronograph. Entrance and exit wounds were immediately documented and measured (Fig. 3).

### Experiments with felt

We quickly became aware that the musket ball was not always producing a clear round hole in the felt material attached to the skull phantom. We thus expanded our initial experiment and fired musket balls at 4 mm industrial felt as well as handmade 3 to 5 mm thick wool felt. Several velocities were experimented aiming to produce a round 19 to 19.5 mm hole into felt. We also utilized a 28 mm cannon to shoot a 25.4 mm steel ball to replicate an iron cartouche ball.

### Radiological imaging

Following the experiments, ballistics phantoms were examined using biplanar digital X-ray (DigitalDiagnost C90, Philips, Netherlands) with pixel size 143 μm and computed tomography (Aquilion One, Toshiba Medical Systems Corp., Tokyo, Japan) with voxel size 500 μm. We aimed to study wound channels and potential remnants of lead inside them.

## Results

**Fig. 4.**
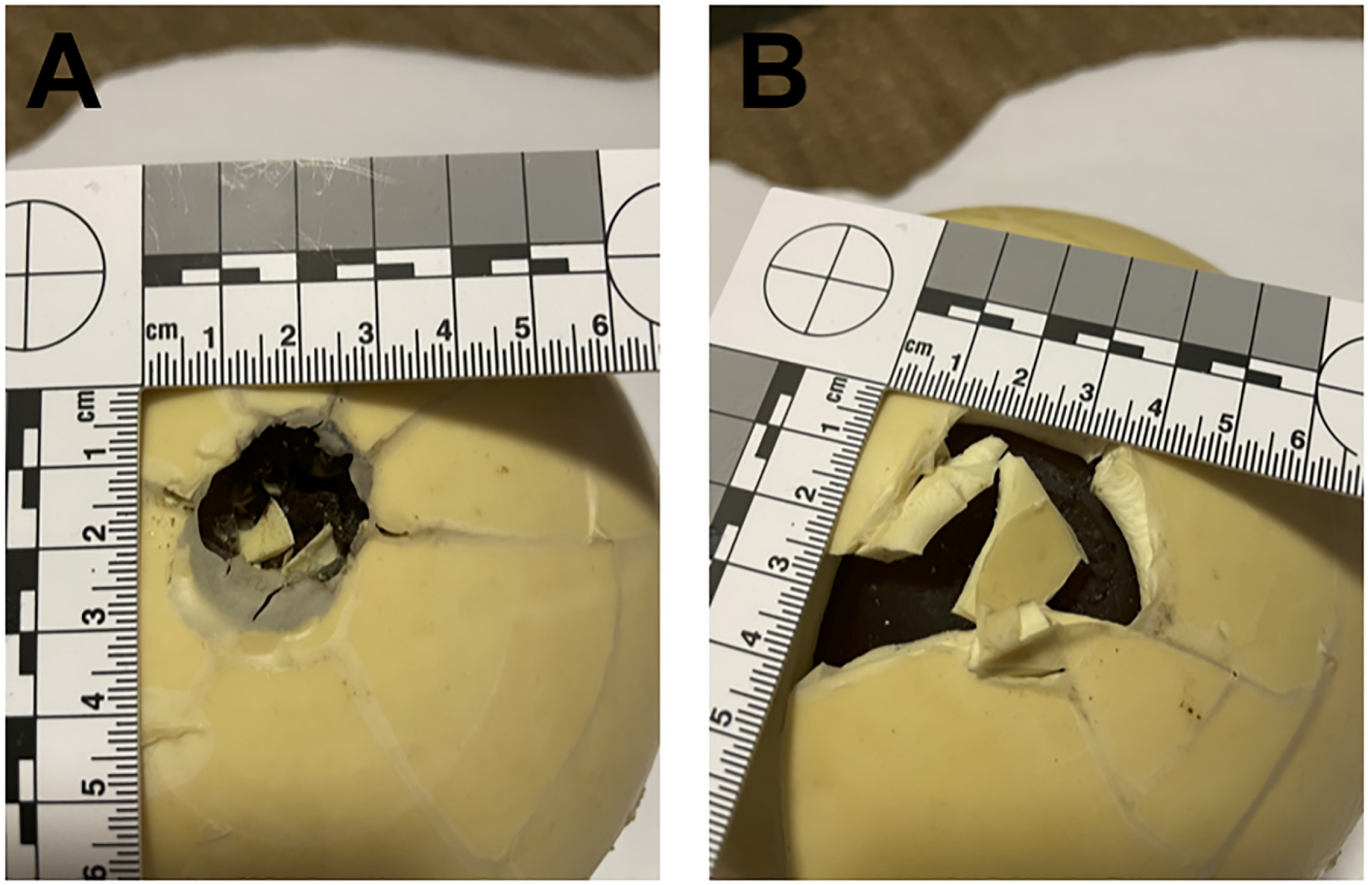
Entrance (A) and exit (B) wounds of experiment 1.

Experiment 1 provided a musket ball velocity of 193 m/s. The ball weight reduced by 0.6 g and its frontal half was clearly deformed. The ball perforated the felt and the phantom. The entrance wound was circular and approximately 21 mm in diameter. The exit wound was more irregular, 25 to 30 mm in diameter (Fig. 4).

Experiment 2 produced a velocity of 194 m/s. The ball deformation was similar to experiment 1. The entrance wound was circular, 20 to 22 mm in diameter. Exit wound was larger, irregular, and 25 to 35 mm in diameter.

**Fig. 5.**
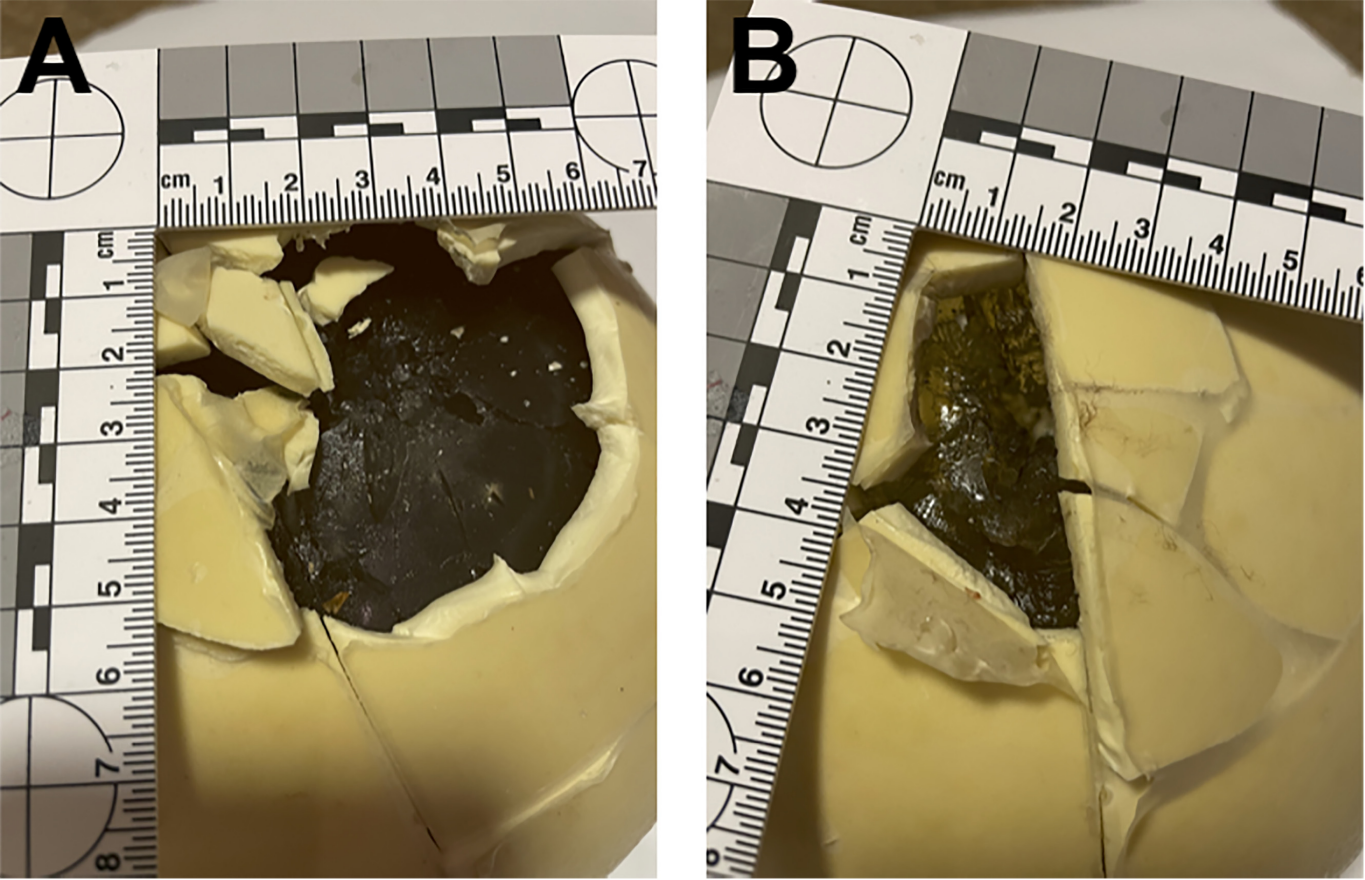
Entrance (A) and (B) exit wounds of the experiment 3.

The ball velocity of experiment 3 was 265 m/s. Ball deformation and weight reduction were similar to the previous experiments. However, the entrance wound was large and irregular with a diameter of 50 to 55 mm. The exit wound, on the other hand, was smaller, just 20 mm in size, but had clear fracture lines in a circular area approximately 50 mm in diameter (Fig. 5).

Experiment 4 produced a velocity of 152 m/s. The ball weight reduced by 0.4 g and the deformation was less visible than in previous shots. The ball perforated through the felt and the phantom, inflicting a circular entrance wound 20 mm in diameter and an irregular exit wound approximately 25 mm in diameter.

**Fig. 6.**
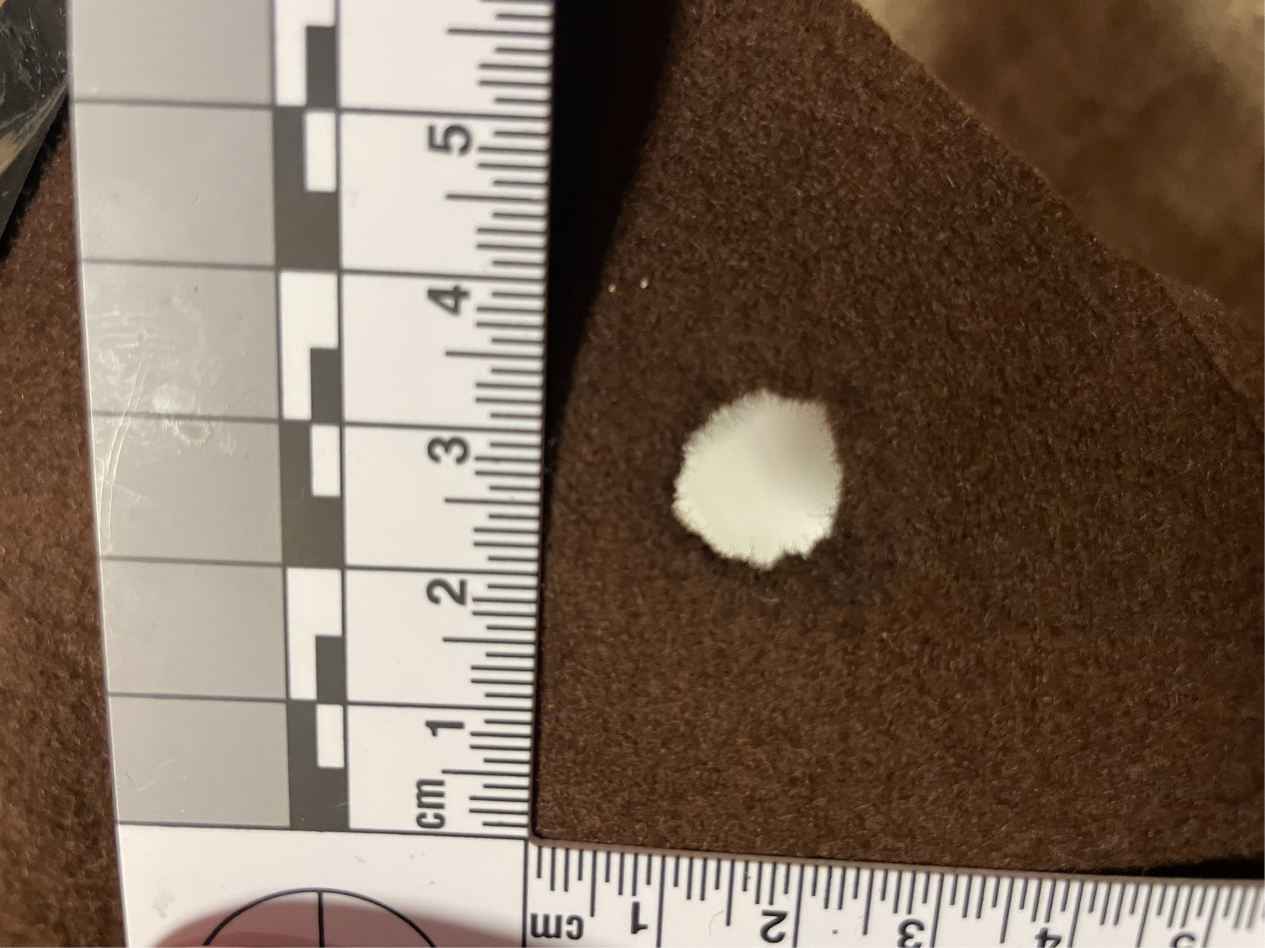
Typical musket ball hole in felt material was approximately 15 mm and largest 17 mm.

**Fig. 7.**
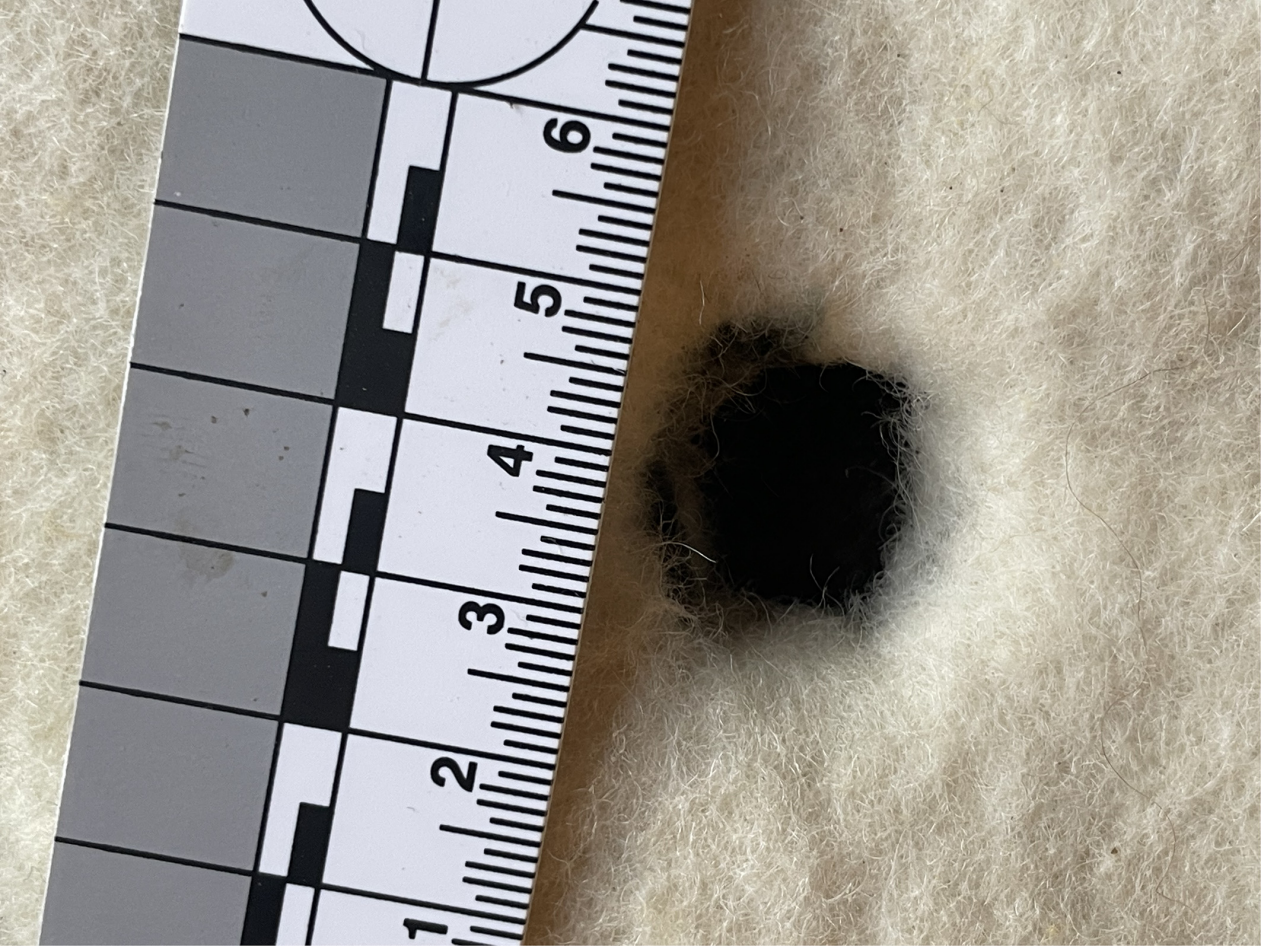
Experiment with a cannon and a 25.4-mm steel ball. With a ball velocity of 214 m/s, the hole was approximately 20 to 21 mm in diameter.

The additional experiments with different felt materials provided interesting results. A 19.5 mm musket ball did not produce 19.5 mm hole. The maximum hole diameter was just 17 mm (Fig. 6) and the average diameter was 15 mm. The hole shape was essentially round but somewhat irregular. Experiments with a cannon and a 25.4 mm ball produced clearly larger, 20 to 21 mm holes (Fig. 7).

**Fig. 8.**
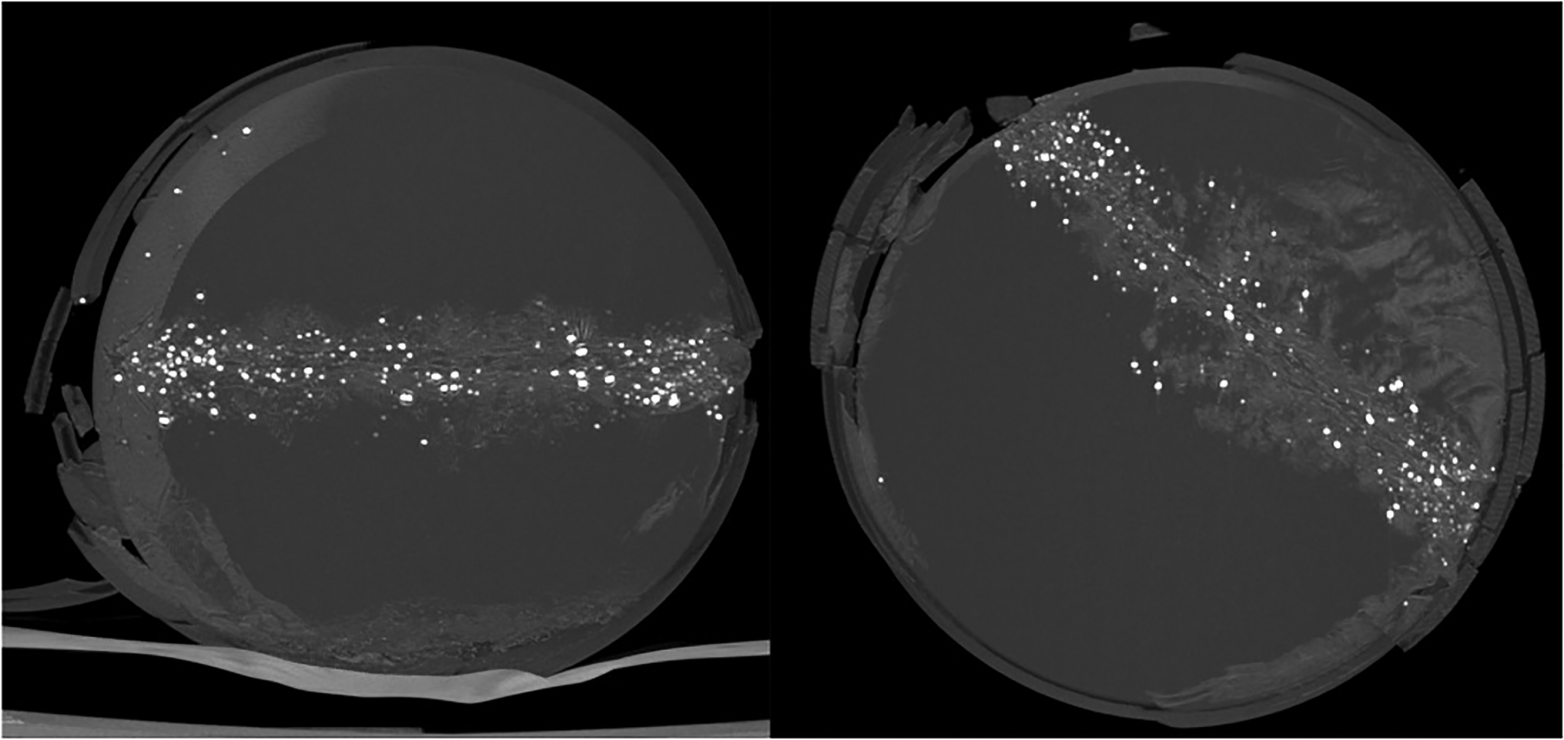
Perpendicular maximum intensity projection CT-reconstructions of a Phantom after experiment 3 showing the clustering of remnants around the entrance and exit holes of a projectile. The entrance hole is on the left.

**Fig. 9.**
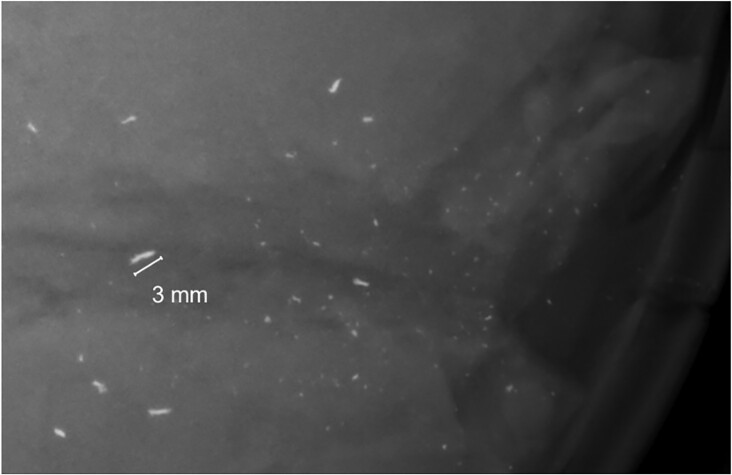
Remnants in a radiograph were typically submillimeter in size. Largest streaky fragments in experiment 3 were around 3 mm in length.

**Fig. 10.**
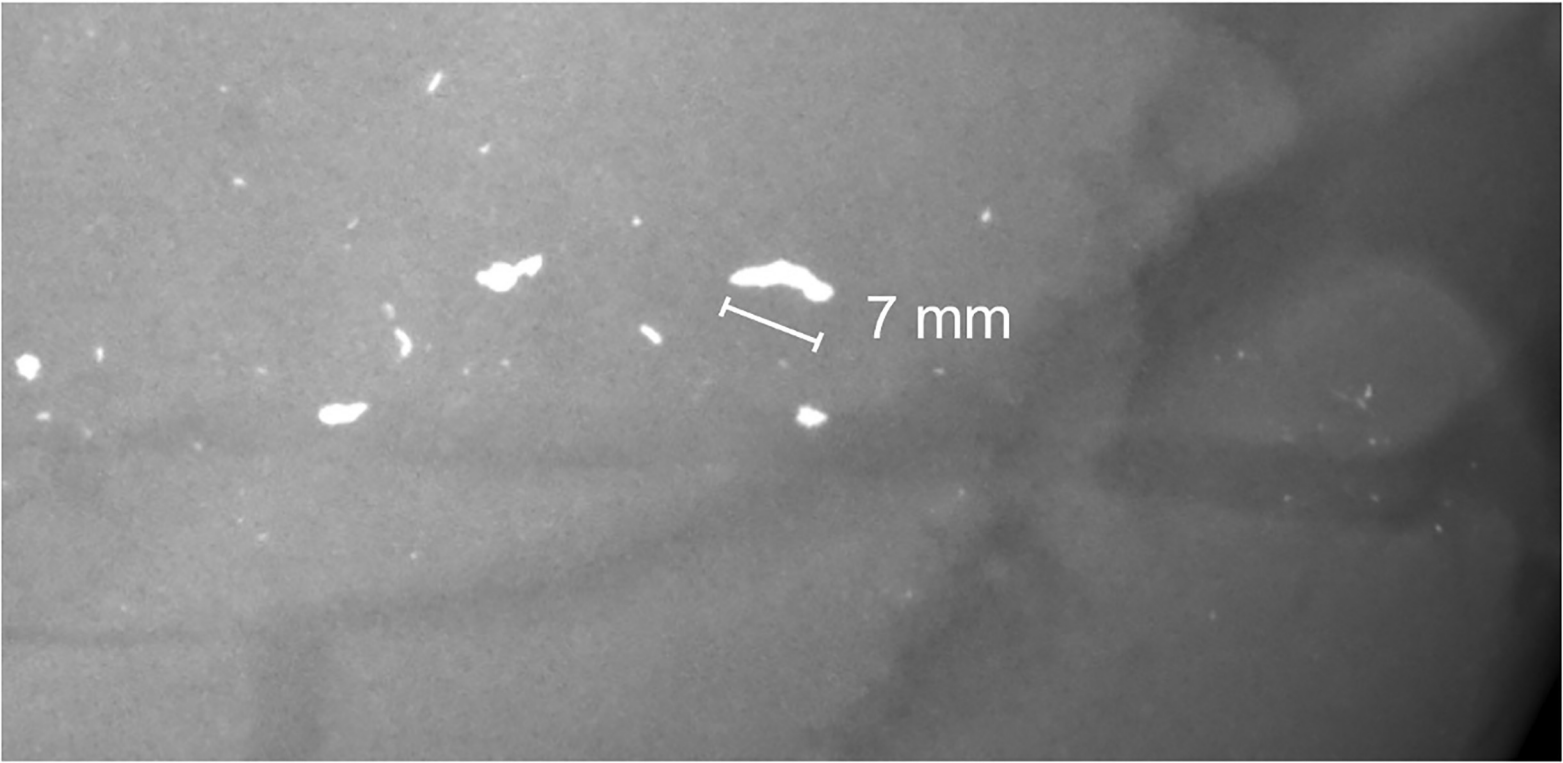
Fragments sized up to 7 mm were seen in the radiograph of the phantom after experiment 1.

Radiological findings were clear and uniform with both biplanar and three-dimensional imaging. Remnants of lead were scattered all over the wound channel. In general, remnants were more concentrated around the entrance, but in the third shot, a significant amount of remnant material was also near the exit. The amount of material was highest, and the size of individual remnants was smallest in the shot with a ball velocity of 265 m/s (Figs. 8 to 10).

## Discussion

Our experiments supported previous conclusions that King Charles XII of Sweden was hit by a projectile that traveled approximately 200 to 250 m/s. Although no remnants of lead were visually observed in the phantoms, lead material was clearly visible in radiography and CT. We could thus conclude that Charles was not killed by a leaden musket ball.

Entrance wounds in experiments 1 and 2 were round in shape and 21 to 22 mm in diameter. Exit wounds were approximately 30 mm in diameter and irregular due to large bone defects. Compared to the entrance and exit wound sizes observed in the 1917 autopsy (1), our experiment produced clearly smaller wounds.

In experiment 3, the entrance wound was much larger than in the two previous experiments. As such, an entrance wound could be larger than an exit wound if the projectile velocity in impact is high enough. In comparison to previous studies experimenting with animal skulls (9), experiment 4 indicated that even the musket ball velocity of under 150 m/s was sufficiently high enough to perforate double felt and the skull phantom.

We could produce morphologically similar but smaller wounds compared to those observed in Charles’ head. The entrance and exit wounds in our experiments were considerably smaller than the ones measured from Charles (1). Although estimating caliber from a defect size is inaccurate (12, 16), our simulation suggests that the projectile in Charles’ case was larger than 19.5 mm in diameter.

One of the most important pieces of evidence regarding projectile size has been the hat that Charles was wearing during his death [e.g. (6)]. A round hole of 19 to 19.5 mm has been widely interpreted as the exact diameter of the projectile. Our experiments demonstrated that the hole in Charles’ hat was probably produced by a projectile significantly larger than 19.5 mm.

The main strengths of this study are the usage of latest ballistic phantoms that provided us with homogenous and reliable test material, and the use of CT for wound characteristics [e.g. (13)]. Our study has also several weaknesses. Apart from excluding lead, we could not confirm the projectile material. We are aware that an iron ball of larger diameter could produce injuries of different patterns and sizes, as it has different terminal ballistic behavior.

According to our findings, the most probable course of events leading to the death of King Charles XII of Sweden would be the following. A ball-shaped projectile that was not leaden, travelled at a velocity of 200 to 250 m/s and perforated through his skull. This velocity would be in line with muzzle velocities of 400 to 500 m/s fired from a distance of approximately 200 m. The round 19.5 mm hole in his hat was at least slightly, but potentially considerably smaller than the diameter of the projectile in question. Larger projectile size is also supported by the size of the bony defects in Charles’ skull.

The utilization of modern techniques has aided the investigation of several historical forensic cases [e.g. (17, 18)]. Our experiment was not an exception, as we could reliably exclude two projectiles, leaden musket ball and the so-called bullet-button. Additional experiments are still needed to clarify the exact diameter and material of the projectile. According to the wound size and shape observed in the 1917 autopsy and the results of our experiments, it appears that the potential projectile diameter would have been larger than 20 mm. According to Clason (19), the smallest iron projectiles of cartouche shot at the time had a diameter of 21.9 mm.

## Supplementary Material

pgac234_Supplemental_File

## Data Availability

All data are included in the manuscript and/or supporting information.
