# Supplementary material for: The death of King Charles XII of Sweden revisited
Source: PNAS Nexus. 2022 Oct 11;1(5):pgac234. doi: 10.1093/pnasnexus/pgac234 (PMC9802245; doi:10.1093/pnasnexus/pgac234)
Supplement: pgac234_Supplemental_File [file pgac234_supplemental_file.docx]

**SUPPORTING INFORMATION**

**Extended methods**

*Shooting experiment*

Altogether 12 shots were fired in this experiment. First four included Synbone ballistic skull phantoms and last eight were only to test different projectiles and velocities on felt hole measurements. Only one type of projectile was used in our skull phantom experiments. A round musket ball in diameter of 19.5mm was selected based on the previous research on topic and especially the19-19,5mm hole in the hat Charles was wearing (Key-Åberg & Stille 1918, Hultkivist 1937). Each musket ball was casted from pure lead and their weight ranged from 43.2 to 43.5 grams.

Remington SP-10 shotgun with 30” barrel length was used to shoot musket balls. A 10 gauge smoothbore shotgun was selected as it provides convenient barrel diameter of 19.7mm in relation to the 19.5mm musket ball in our test. An empty 10/89 shotgun shell with shotgun primer was handloaded with Hodgdon Pyrodex RS black powder substitute and 19.5mm musket ball. A piece of paper towel was squeezed on the top of the powder charge to act as a wad between the powder and the musket ball. Musket ball and powder charge weight were measured with Hornady G2 1500 electronic scale. The musket ball weight prior and after the experiment was recorded to nearest 0.1g.

We aimed to provide three speed categories according to previous research that has speculated on potential projectile velocity in impact (Hultkvist 1937, Grenander 1988). We were mostly interested in the speed category of approximately 200m/s as that would be a realistic velocity for musket ball fired from the fortress of Fredriksten some 200 meters from the death scene. Previous research has also suggested that the velocity of the projectile would have been at least 150m/s but clearly less than 250m/s (Hultkvist 1937).

Three different powder loads were selected to achieve musket ball velocities of 150, 200 and over 250 meters per second. To achieve the velocity of 150m/s we used a powder load of 2.4g, for 200m/s the load was 3.11 g, and for over 250m/s 4.5g, respectively. A Caldwell Ballistic Precision chronograph was used to measure the actual musket ball velocities. With these velocities we aimed to replicate musket shots taken from a range of approximately 200 meters, the distance between enemy lines and Charles during the night he died.

Test firing was performed at an enclosed shooting range following adequate safety measures. In total four shots were fired towards four individual skull phantoms thus providing us with four separate experiments. Each phantom was positioned on a solid, wooden platform. Shots were taken from a distance of five meters. Entrance and exit wounds were immediately examined and measured. A Caldwell chronograph was placed directly in front of the phantom to measure the projectile velocity prior the hit. As we were interested in recovering the musket ball after each shot, sawdust bales were placed right behind the skull phantom to reduce the speed and eventually stop the projectile.

*Experiments with felt*

Already at the very beginning of our experiment we became aware that the musket ball was not always producing a clear and round hole into the felt material that was attached to the skull phantom. We thus expanded our initial experiment and fired musket balls at 4mm industrial felt as well as handmade 3-5mm thick wool felt. Several musket ball velocities were experimented (four additional shots with velocities ranging from 114 m/s to 286 m/s) aiming to produce a round 19-19,5mm hole into felt. In addition we utilized a 28mm cannon to shoot 25,4mm steel ball to replicate an iron cartouche ball. Four shots with muzzle velocities between 214 and 380m/s were taken to demonstrate the potential of this larger projectile.
